# The First Case of a Homozygous CCNO NM 021147.4 Mutation Associated With Primary Ciliary Dyskinesia in Two Indian Siblings

**DOI:** 10.7759/cureus.52237

**Published:** 2024-01-13

**Authors:** Ola Alhalabi, Atqah Abdulwahab, Merlin Thomas

**Affiliations:** 1 Pediatric Pulmonology, Sidra Medicine, Doha, QAT; 2 Pulmonology, Hamad General Hospital, Doha, QAT

**Keywords:** encoding cyclin o (ccno), reduced generation of multiple motile cilia (rgmc), chronic sinopulmonary disease, indian, primary ciliary dyskinesia (pcd)

## Abstract

Primary ciliary dyskinesia (PCD) is a heterogeneous autosomal recessive disease marked by organ lateralization in 50% of patients, chronic sinopulmonary disease, infertility in men, and neonatal respiratory distress.

Respiratory control cells contain *CCNO *in their apical cytoplasm, which is necessary for the development of multiciliate cells, basal body amplification, and migration. Reduced generation of multiple motile cilia, a rare form of PCD, has been linked to *CCNO *gene abnormalities*. *Individuals with *CCNO* mutations have been reported to suffer from severe lower respiratory infections that cause progressive impairment of lung function. For the first time, we describe the *CCNO NM 021147.4* (c.258 262dup.p, Gln88argfs*8 Homozygous) gene mutation in an Indian consanguineous family that resulted in severe PCD.

## Introduction

Primary ciliary dyskinesia (PCD), an autosomal recessive condition characterized by motile cilia malfunction, displays clinical and genetic variation. Some of the clinical symptoms of PCD include left-right lateralization, infertility, and chronic upper and lower respiratory illness [1-5]. There is no one best way to diagnose PCD; instead, a variety of methods can be used, such as a combination of nasal nitric oxide (nNO), high-speed genetic analysis, immunological fluorescence of ciliated cells, transmission electron microscopy (TEM), high-speed video microscopy analysis, immune fluorescence of ciliated cells, and genetic analysis (gene panel analysis or extensive genetic analysis) [6,7].

There is a significant correlation between phenotype and specific genetic changes. Reduced generation of multiple motile cilia (RGMC) has been linked to *CCNO *gene mutations, which are also more likely to have a more severe respiratory disease phenotype with pulmonary failure at a younger age [8].

For the first time, we describe the *CCNO NM 021147.4* (c.258 262dup.p, Gln88argfs*8 homozygous) gene mutation that caused severe PCD in a consanguineous Indian family.

This article was previously presented as a meeting abstract at the 2022 American Thoracic Society International Conference Meeting on May 13-18, 2022.

## Case presentation

Our case study focuses on two consanguineous sisters from India who visited the Pediatric Pulmonary Department at Sidra Hospital in Qatar when they were 17 and 15 years old. They presented with chronic wet cough and were found to have progressive loss of lung function that eventually led to end-stage lung disease and the requirement for lung transplantation in the case of the elder sister. Since infancy, both siblings had suffered significant lower respiratory infections, chronic rhinorrhea, and recurrent ear infections.

**Figure 1:**
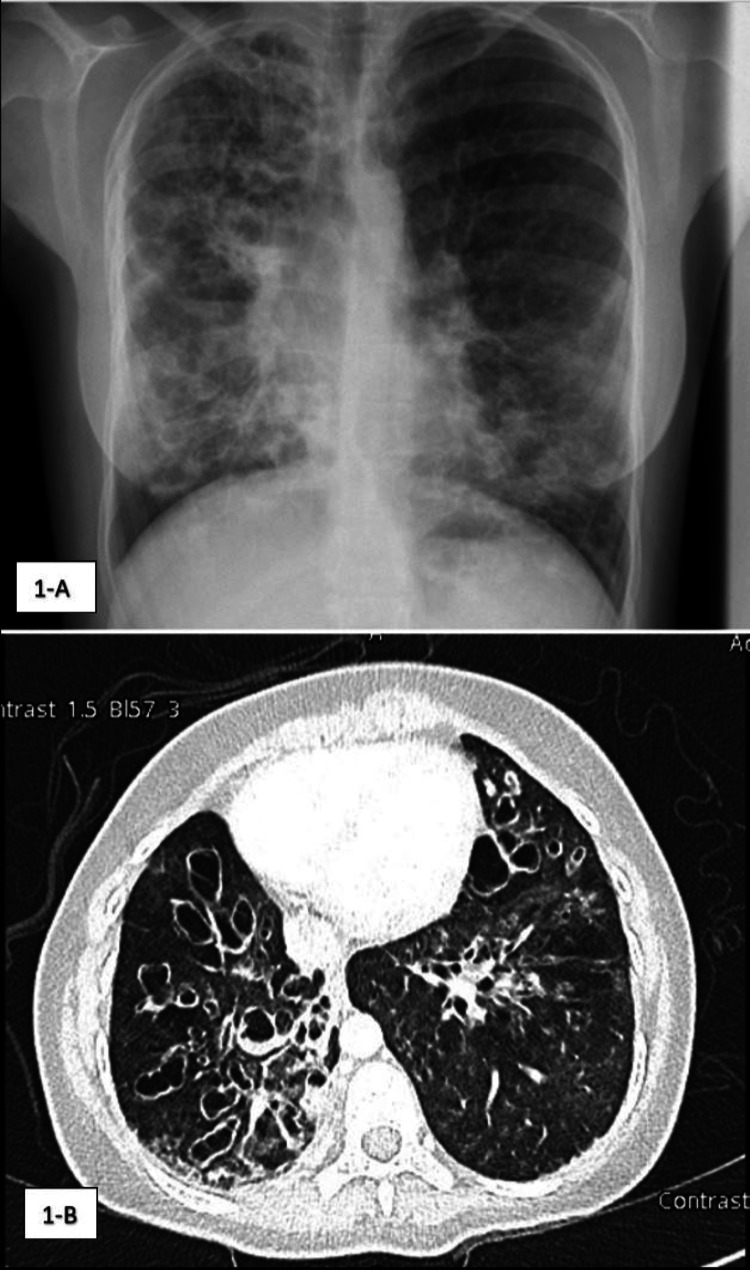
Chest X-ray and CT chest findings of the elder sister. A: Anteroposterior chest X-ray. B: CT chest, axial view, lung window. Chest radiographical imaging of the elder sister showing bilateral varicose bronchiectasis worse on the right side.

**Figure 2:**
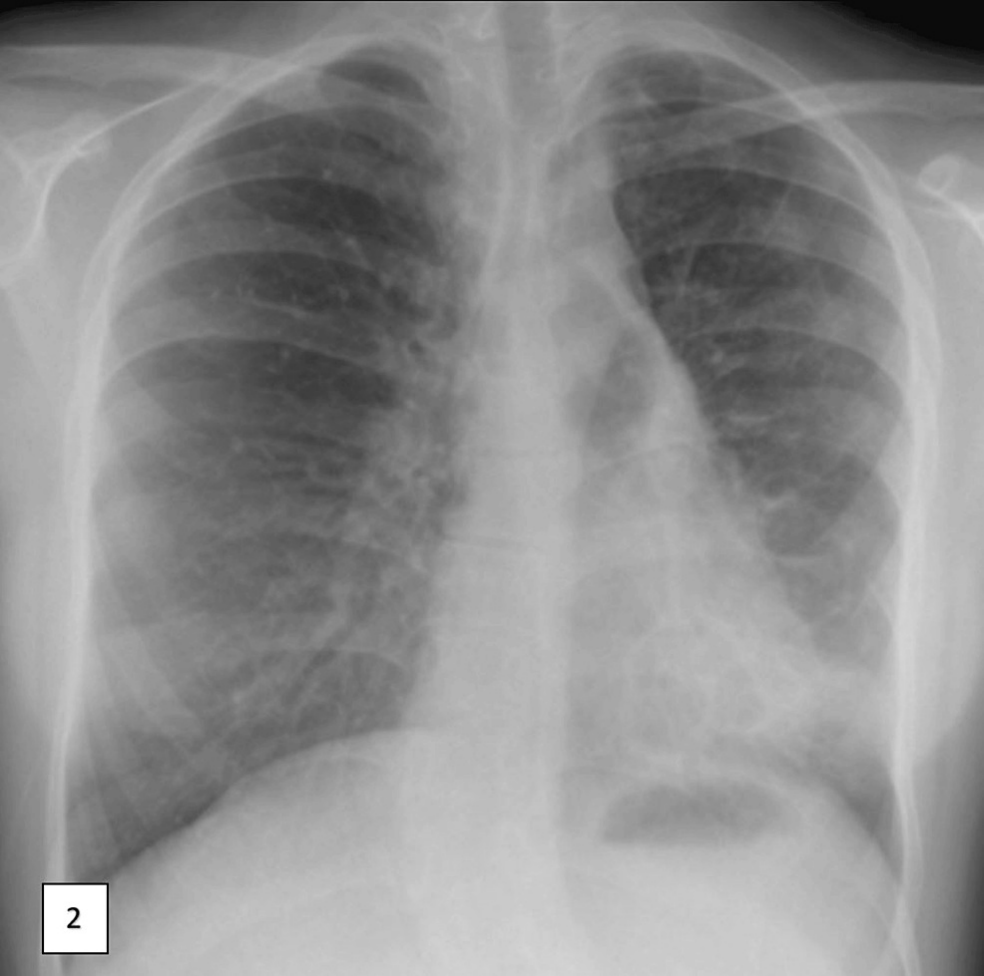
Anteroposterior chest X-ray of the younger sister. Chest radiography showing left lower lobe consolidation.

**Figure 3:**
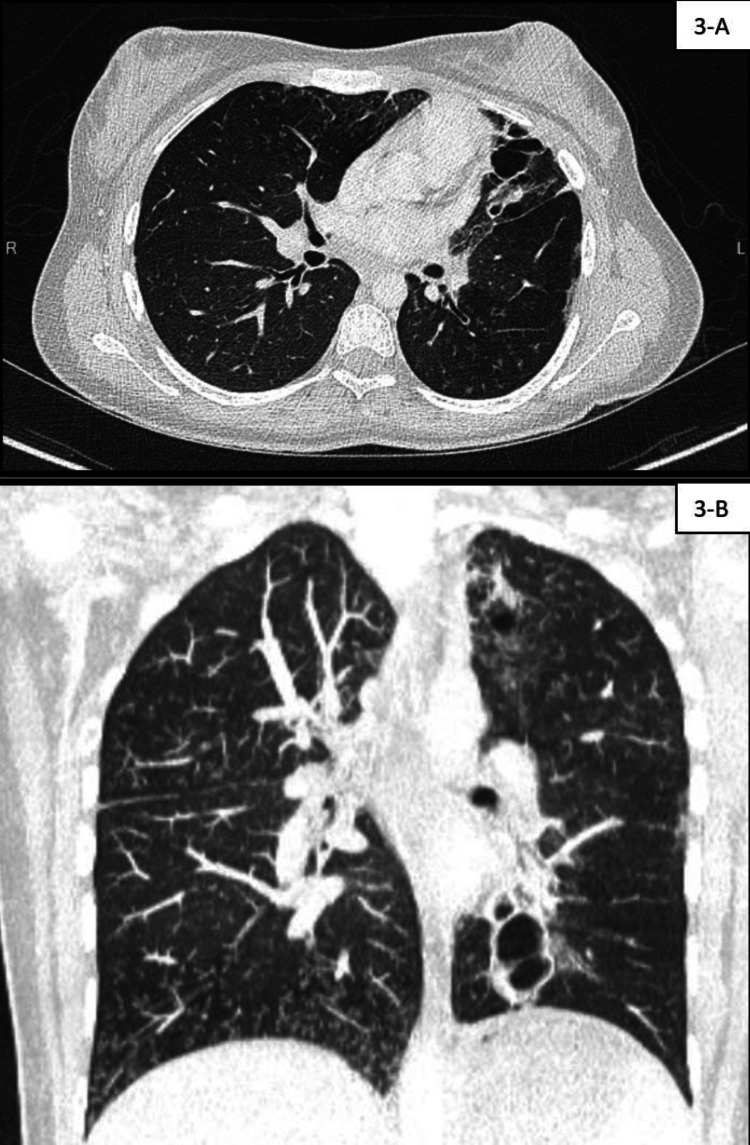
CT chest findings of the younger sister. A: CT chest, axial view, lung window. B: CT chest, coronal view, lung window. CT of the chest shows decreased volume of the left upper lobe and lingula with diffuse cystic bronchiectasis. There is also bronchiectasis within the left lower lobe with a mucus plug and hyperinflation of the right lung with diffuse tree-in-bud changes of the right middle lobe likely secondary to infection.

Bronchiectasis was detected by computed tomography (CT) of the chest when the elder sister was 14 years old (Figure 1) and the younger sister was seven years old (Figures 2, 3).

**Figure 4:**
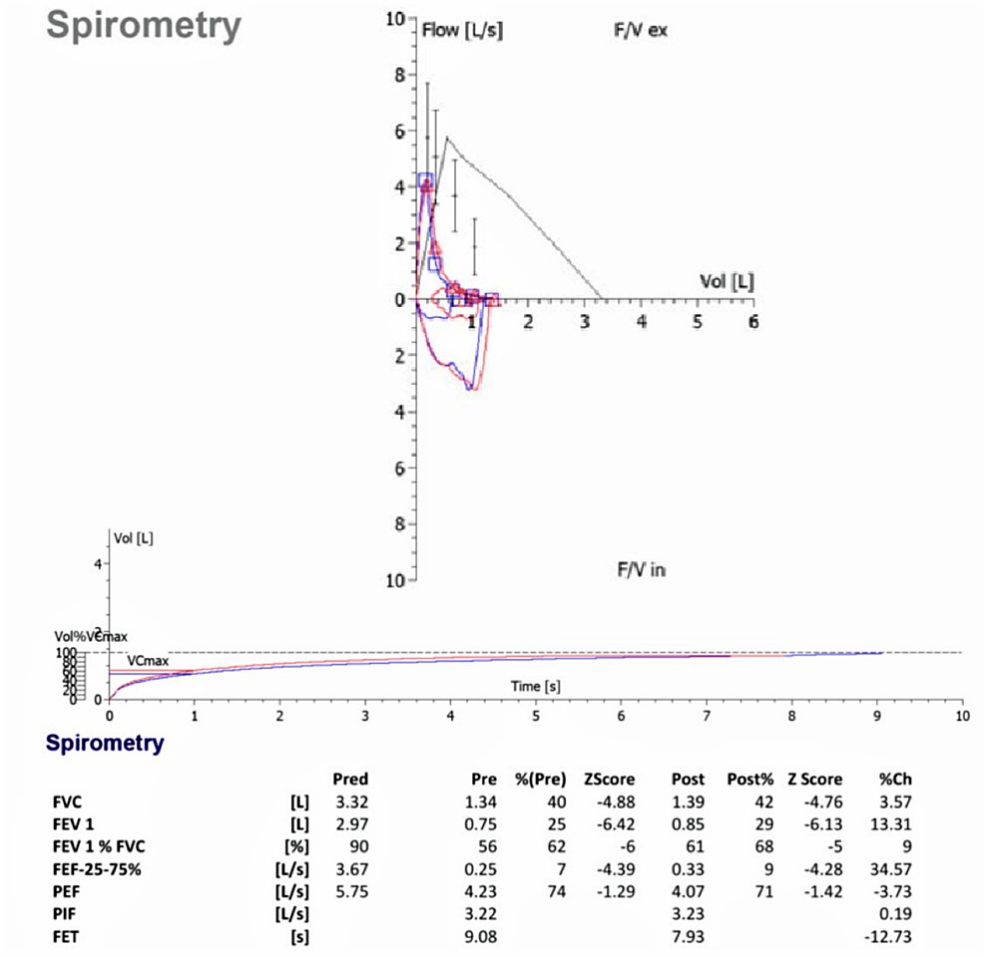
Spirometry findings of the elder sister. Spirometric measurement of the elder sister showed mixed restrictive and obstructive airway disease. FVC = forced vital capacity; FEV = forced expiratory volume; PEF = peak expiratory flow; PIF = peak inspiratory flow; FET = forced expiratory time

**Figure 5:**
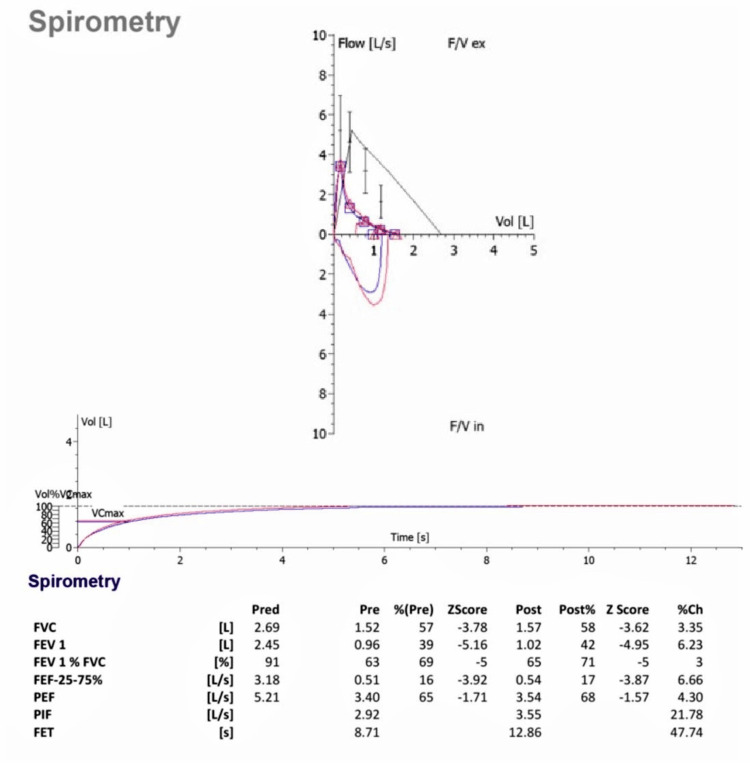
Spirometry findings of the younger sister. Spirometric measurement of the younger sister showed mixed restrictive and obstructive airway disease. FVC = forced vital capacity; FEV = forced expiratory volume; PEF = peak expiratory flow; PIF = peak inspiratory flow; FET = forced expiratory time

Physical examination was pertinent for bilateral crackles and clubbing in both sisters. The elder sister was hypoxic requiring 1‐2 L of oxygen per minute via a nasal cannula. Expiratory flow volume (spirometry) revealed significant mixed restrictive and obstructive airway disease, which was worse in the elder sibling. The forced expiratory volume in one second (FEV1) and forced vital capacity (FVC) in the elder sister were 25% and 40% predicted, respectively (Figure 4). Whereas the FEV1 and FVC in the younger sister were 39% and 57% predicted, respectively (Figure 5).

Immune deficiency and cystic fibrosis were ruled out by blood tests and sweat chloride measurements. Both sisters had low nNO levels. The elder sister’s nNO was 63.3 ppb (predicted 200‐1,000) and the younger sister’s nNO was 88.3 ppb. PCD was diagnosed based on clinical phenotype and low nNO levels, which were further confirmed through genetic sequence analysis and deletion/duplication for PCD, which revealed the pathogenic variant *CCNO NM 021147.4* (c.258 262dup.p, Gln88argfs*8 homozygous) gene mutation.

## Discussion

We described two Indian sisters who experienced early-onset respiratory symptoms, obstructive ventilatory dysfunction, and situs solitus. They were identified as having PCD based on the full exon of the CCNO gene and low nNO levels.

Patients with ciliogenesis-related mutations in the genes *CCNO *and *MCIDAS *have been reported to have a worse phenotype than those with other kinds of PCD, which is consistent with the current cases [8]. There are currently 50 known PCD genes that cause a variety of functional defects ranging from abnormal beat patterns through impairment of dynein arms to a complete absence of cilia [9,10]. Although nNO showed good diagnostic accuracy as a PCD diagnostic test when compared to the extended reference standard of TEM and/or genetic testing [11], it is crucial to confirm the genetic diagnosis, given the present evidence of links between genotype and phenotype [12].

Patients with PCD, which is secondary to *CCNO *mutation, have early-onset respiratory symptoms, recurrent lower respiratory tract infections, and progressive loss of lung function, which is consistent with our cases. Situs solitus, like in the cases presented, has only been documented in people with *CCNO *mutations [8]. The other documented symptoms are recurrent pneumonia, sinusitis, and otitis media (Table 1).

**Table 1:** CCNO gene mutation studies.

Study	Number of cases	Genetic	Phenotype	Nasal NO	Ultrastructural defect	Video microscopy
Wallmeier et al. (2014) [8]	16	The study discovered homozygous loss-of-function mutations (p.Gly85Cysfs*10) in CCNO using whole-exome sequencing	After birth, 12 out of 16 babies experienced respiratory distress. One of the women was infertile. Everyone exhibited situs solitus, bronchitis, and recurrent infections of the upper and lower respiratory tracts. At the age of 34, two people who had terminal respiratory failure underwent lung transplants	Not done	All affected people either had no cilia at all or had a significant reduction in cilia	Respiratory epithelial cells showed a marked reduction in the number of multiple motile cilia (MMC) covering the cell surface. The few residual cilia that correctly expressed axonemal motor proteins were motile and did not exhibit obvious beating defects
Kumar et al. (2015) [12]	80	Unavailable	The mean age of presentation was 9.6 (range = 2–15) years. Overall, 62.5% of the population was younger than 5 years old. Clubbing was present in 58 (72.5%) children	Not done	Not done	Not done
Casey et al. 2015 [13]	5	A novel 1 bp duplication in RSPH4A. CCNO, KCNN3, and CDKN1C.a ∼3.5-kb deletion in DYX1C1	Recurrent lower respiratory tract infection: 5/5. Bronchiectasis on CT of the thorax: 5/5. Hearing loss in 2/5 patients. 2/5 of the patients had early-onset severe cardiomyopathy, type III glycogen storage disease, and developmental delay. Neonatal pneumonia affected 1/5 of patients. Recurrent otitis media in 2/5	Repeat nasal oxide screening tests consistently yielded low results of 30 to 50 ppb	A displacement of one of the peripheral doublets was seen in certain cilia, with 22% of them lacking the central pair. Both the outer and inner dynein arms were normal. Ciliary aplasia was present in 2 instances. In one instance, both the inner and outer dynein arms were absent	All of the cilia were abnormally static or dyskinetic. Although the pattern was incomplete and the cilia seemed stiff without cleaning any material, it was still possible to see the cilia moving when viewed from above
Amirav et al. 2016 [14]	91	In 15 people (16%), biallelic CCNO mutations were found. Three compound heterozygous mutations and seven homozygous mutations were found. Every single identified mutation was inherited autosomally recessively within the families. Three frameshift mutations (c.262263dupGGCCC, p.Gln88Argfs8; c.165delC, p.Gly56Alafs38; c.481482delCT, p.Leu161Glyfs73), one missense mutation (c.638T>C, p.Leu213Pro), and one deletion mutation were found	The age of affected individuals ranged from 5 to 54 years. At a median age of 20 years, the initial genetic diagnosis was made. All situs solitus 11/14 cases (85%) of neonatal respiratory distress syndrome. Otitis media recurrent in 10/15 (67%) people, and 13/14 (93%) people had sinusitis. Documentation of bronchiectasis by radiographic imaging in 13/14 (93%). At the age of 43, lung transplantation was done. The composition of the situs was normal in all those with CCNO mutations. One affected female underwent assisted reproduction using in vitro fertilization to become pregnant. One affected male fathered a child without medical assistance. One individual with CCNO mutation had arrested hydrocephalus	Two people showed nasal NO readings that were within the normal range. Mean nasal NO values of 50.32 ± 68.62 nL/minute (14 individuals)	The transmission electron microscopy of three people revealed normal microvilli composition, but the basal bodies, specialized centrioles that initiate ciliary axonemes in the apical regions of respiratory epithelial cells, were absent or significantly diminished. Displaced basal bodies and rootlets (propagating from the basal body) were found in the cytoplasm of some respiratory epithelial cells	
Henriques et al. 2021 [15]	3	Heterozygous mutation, CCNO gene c.253_257GGCCC(3)(p.Gln88fs) and c.263_267dup(p.Val90fs) homozygous mutation, c.263_267dup(p.Val90fs) and c.263_267dup(p.Val90fs)	3/3 respiratory symptoms that started early. Situs solitus. Due to lung collapse, one required a lobectomy. One patient had two tympanostomy tube insertions due to recurrent otitis media with effusion and conductive hearing loss	Not done	Reduced or absent number of cilia, and normal ultrastructure in residual cili.	Most epithelial cells with bald epithelium, residual cilia with uncoordinated ciliary beat frequency and pattern
Emiralioğlu et al. 2019 [16]	46	DNAH5, CCDC40, RSPH4A, DNAH11, HYDIN, CCNO, DNAI1, ARMC4, TTC25, DNAH1, and CCDC39 gene	The median age at diagnosis (median: 3 years; range, 6 months to 4 years). 44 patients had rhinitis, whereas 41 had newborn respiratory distress. The sinusitis returned in 36. 14 people had recurrent otitis. Six were hard of hearing. Clubbing was seen in seven. Fourteen had a total inversus situation. Six (atrial septal defect, patent ductus arteriosus, and mitral valve prolapse) had congenital cardiac defects. Four patients had Lobectomy. Four patients had undergone ear, nose, and throat surgery	Median nasal NO was 8 ppb (minimum: 5, maximum: 40)	Seven patients had a nasal biopsy: three had outer dynein arm defect. Two microtubule disorganizations with the inner dynein arm. Two had central pair abnormality	Cilia were hypokinetic in 28 individuals. Four exhibited hyperkinetic cilia. Twelve had stiff patterns. Two had abnormal circular movement
Guan et al. 2021 [17]	75	DNAH11 variants (15 individuals). DNAH5 variants (nine individuals), CCDC39 variants (five individuals), DNAH1 variants (four individuals), CCNO variants (three individuals), DNAI1, HEATR2, RSPH9, or DNAAF3 (two individuals for each). CCDC40, LRRC6, SPAG1, ARMC4, RSPH4A, CCDC114, and DNAH14 mutated in one individual each	Median age at diagnosis was 7.0 years (range = 2 months to 14 years). A chronic wet cough affected 66 out of 75 people. 58/75 people had recurrent sinusitis. 57/75 people had bronchiectasis. Respiratory distress in newborns was present in 30% of the cases. There were 6/75 cases of postinfectious bronchiolitis obliterans as the first presentation, while 21/75 patients had coexisting asthma	Not done	(8/50) outer dynein arm (ODA) defects. Inner dynein arm (IDA) defects in conjunction with central apparatus (CA) defects and microtubule disorganization (MTD) were classified as IDA/CA/MTD. (8/50) had IDA defects, CA defects, and MTD. (12/50) ODA and IDA. (10/50) CA or IDA defects. (4/50 ) Oligocilia. (5/50) Normal structure	Not done

There are no reports of genetic PCD in India, and only one publication stated that patients with recurrent sinusitis, otitis, and pneumonia in India who also had a fractional exhaled NO level under 10 ppb and who had not undergone genetic testing were likely to have PCD [13]. To our knowledge, this is India’s first report on PCD with *CCNO *which accounts for two out of 318 cases that have been published in the literature (Table 1).

In 16 people who had the first *CCNO *mutation in 2014, a malfunction in the mother centriole formation and migration at a late stage of multiple motile cilia (MMC) differentiation led to a significantly decreased number of MMCs. Congenital mucociliary clearance disorder with RGMC is used to denote this hereditary condition [8]. Following the study, there have since been several *CCNO *reports (Table 1). In a study of five PCD patients from three different Irish traveler families, it was discovered that a sibling pair in Irish family B had the *CCNO *gene [14]. In another investigation, researchers looked for *CCNO *mutations in 170 Israeli families with mucociliary clearance disorders and identified two novel variations (p.Gly56Alafs38; c.165delC, c.638T>C, p.Leu213Pro), and two known mutations were found in 15 individuals from 10 families (6% prevalence) [15]. In Lisbon, Portugal, 12 patients underwent PCD genetic testing confirming the diagnosis, with three presenting *CCNO *mutations [16]. In Turkey, out of a total of 265 patients with PCD during a five-year period, 46 had genetically determined PCD using whole-exome sequencing at a single facility, and four had *CCNO *[17]. There have been multiple publications from China. One of these described 58 individuals with PCD, 51 with hereditary PCD, and three with *CCNO *[18]. This presenting case study is not a meta-analysis, and its limitation arises from the small number of patients from India, which has not been previously explored in the current literature.

## Conclusions

PCD with *CCNO *mutations is a relatively rare disease. Our findings highlight the significance of considering PCD based on *CCNO *mutations in people with situs solitus to have a more severe respiratory disease phenotype with lung failure at earlier ages. It is also critical to include individuals from various racial and ethnic origins in PCD-associated genetic *CCNO *mutation.
